# Imaging features of myopericytoma arising from the parotid gland: Report of 2 cases and literature review

**DOI:** 10.1097/MD.0000000000025471

**Published:** 2021-04-09

**Authors:** Yao Pan, Lu Chen, Dan Shi, Ying Chen, Ri-Sheng Yu

**Affiliations:** a Department of Radiology, The Second Affiliated Hospital, Zhejiang University School of Medicine, Jiefang Road 88#, Hangzhou 310009; b Department of Radiology, The Fourth Affiliated Hospital, Zhejiang University School of Medicine, Shangcheng Avenue N1#, Yiwu 322000, China.

**Keywords:** magnetic resonance imaging, myopericytoma, parotid gland

## Abstract

**Rationale:**

Myopericytoma of the parotid gland is a rare condition of which preoperative definitive diagnosis is relatively challenging. The purpose of this case report is to highlight the radiologic features of myopericytoma of parotid gland.

**Patient concerns:**

A 62-year-old man presented with a history of a walnut-size mass in left parotid gland when yawned for half-month, and a 48-year-old woman complaint about a grape-size, painless mass behind the right ear for a month.

**Diagnoses:**

Radiological examinations suggested that both lesions were cyst-solid mixed lesions with relatively smoothed margins, with or without significant enhancement while the lesion without enhancement had a hemorrhage. Then a diagnosis of benign tumor arising from the parotid gland was made. Final diagnosis of myopericytoma was confirmed by histopathological and immunohistochemical examinations after surgical resection.

**Interventions:**

Both patients underwent excision of the tumor and the superficial parotidectomy with facial nerve preservation.

**Outcomes:**

Both patients recovered without any intraoperative or postoperative complication and had no signs of recurrence during a 17-month and 5-year follow-up.

**Lessons:**

Parotid gland myopericytoma is an exceedingly rare tumor which diagnosis can be challenging, and this is the first published report specifying the magnetic resonance features of the disease.

## 1 Introduction

The term myopericytoma was first proposed by Requena et al as an alternative designation for solitary myofibroma derived from myopericytes.^[1]^ Myopericytoma describes a benign usually subcutaneous tumor that is composed of myoid-appearing oval to spindle-shaped cells with a striking tendency for concentric perivascular growth. The concept of perivascular myoid differentiation was established by Granter.^[2]^ In 2002, the World Health Organization officialized the term “myopericytoma” for use in clinical diagnoses.^[3]^ Myopericytoma arises most commonly in middle adulthood, but may also occur in child.^[4]^ Myopericytoma can be multifocal involving single or multiple anatomic regions,^[5]^ and tends to occur predominantly in the skin and superficial soft tissue of the distal extremities (hand, foot, ankle, and leg), followed by the head and neck region, and the trunk.^[6]^ Myopericytoma of the parotid gland is very rare. To date, only 5 published cases of myopericytoma arising from the parotid gland have been reported worldwide, and only 1 report described the computed tomography (CT) imaging features in detail. Current report provides a comprehensive description of parotid gland myopericytoma, including its complete clinical course and imaging findings. Reviews of past literature on this rare condition were also discussed.

### 1.1 Consent

The retrospective case report was approved by both patients, as well as the ethics committee of The Second Affiliated Hospital, Zhejiang University School of Medicine.

## 2 Case reports

### 2.1 Case one

**Figure 1:**
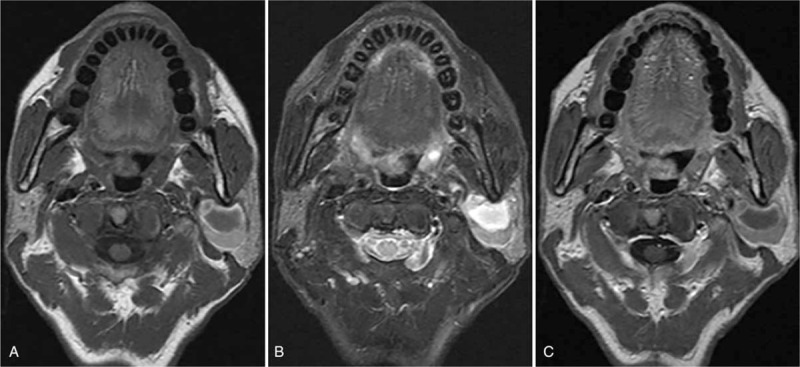
A 62-yr-old man with myopericytoma in the left parotid gland. (A and B) T1-weighted images (A) and T2-weighted images (B) revealing a well defined, cyst-solid mixed mass with slightly high signal intensity in the solid portion; (C) Axial enhanced scan revealing no enhancement of the lesion. The areas of necrosis and cystic degeneration were non-enhanced.

A 62-year-old man was admitted to our hospital following the discovery of a walnut-size mass in left parotid gland when yawned for half a month without any clinical symptoms. His medical history was otherwise unremarkable. Physical examination revealed a soft, painless, well circumscribed, and mobile mass in the left parotid region. Facial nerve function of the patient was normal on both sides. Ultrasound revealed an oval-shape, cyst-solid mixed, 3.0 cm × 2.0 cm mass located in the left parotid gland. Magnetic resonance imaging (MRI) showed a cyst-solid mixed, clearly defined mass in the left parotid gland. The solid component exhibits slightly high signal both on T1-weighted images and T2 weighted images, indicating the possibility of hemorrhage. The area of cystoid variation showed low signal with peripheral high signal on T1-weighted images and high signal on T2-weighted images (Fig. 1A and B). Gadolinium-enhanced MR imaging detected no enhancement (Fig. 1C).

**Figure 2:**
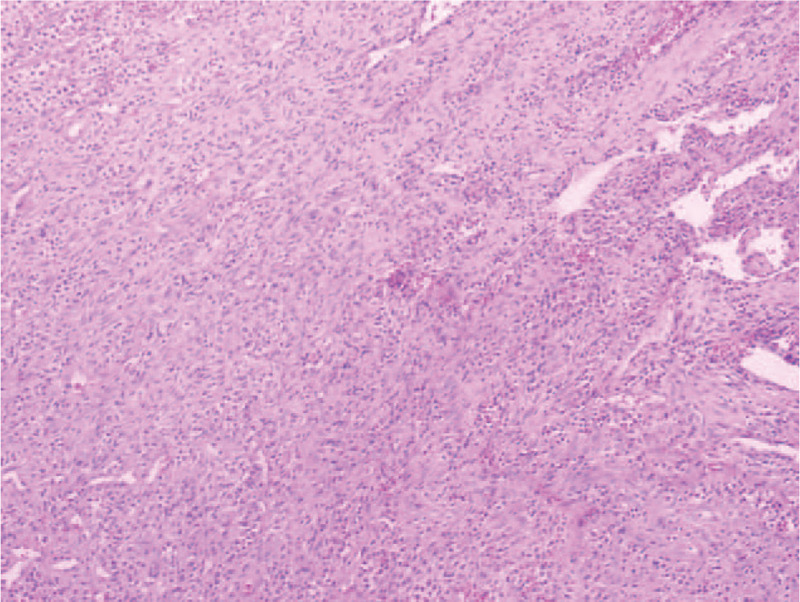
Photomicrograph of histology specimen showed numerous thin-walled vessels surrounded concentrically by proliferative spindle-shaped myoid tumor cells in peripheral zone with obviously hemorrhage and necrosis. (Hematoxylin-eosin stain, original magnification, 100×.)

Surgical excision of the superficial lobe of the left parotid gland and the tumor was performed. All facial nerve branches were preserved. The tumor was in the deep lobe and had invasion of adjacent tissue. The gross appearance of the resected specimen for the mass showed a soft, clear margined tumor of 2.3 cm × 2.0 cm × 3.0 cm in size and the content was black-brown. Photomicrograph of histology specimen showed numerous thin-walled vessels surrounded concentrically by proliferative spindle-shaped myoid tumor cells in peripheral zone (Fig. 2). Hemorrhage and necrosis in the nodule were evident. No malignant features such as nuclear anaplasia, increased mitoses, or infiltrative growth were found in the specimen. Immunohistochemical staining was positive for smooth muscle actin (SMA). The cluster of differentiation 34 (CD34) immunoperoxidase stain only decorated the endothelium of vessels, but the perivascular concentric myoid tumor cells were not immunoreactive. Based on these findings, this tumor was diagnosed as a benign myopericytoma arose from the parotid gland.

After 17 months of follow-up, the patient had no signs of recurrence.

#### 2.1.1 Case two

**Figure 3:**
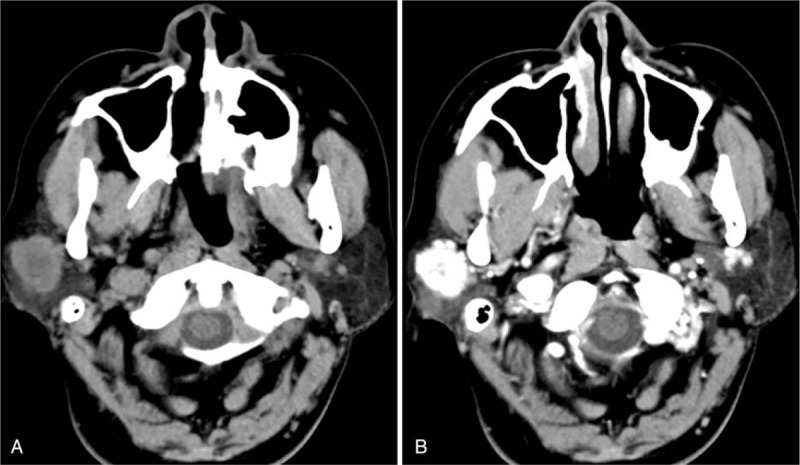
A 48-yr-old woman with myopericytoma in the right parotid gland. (A) Pre-contrast CT image revealing a 2.2 cm × 1.4 cm, relatively well defined, heterogeneous iso-density mass with central lower density in the right parotid gland; (B) Enhanced scan revealing mass with significantly heterogeneous enhancement and central irregular non-enhanced area. The CT values of the significant-enhanced areas in pre-contrast and enhanced scans were 44.82 HU and 157.32 HU, respectively.

**Figure 4:**
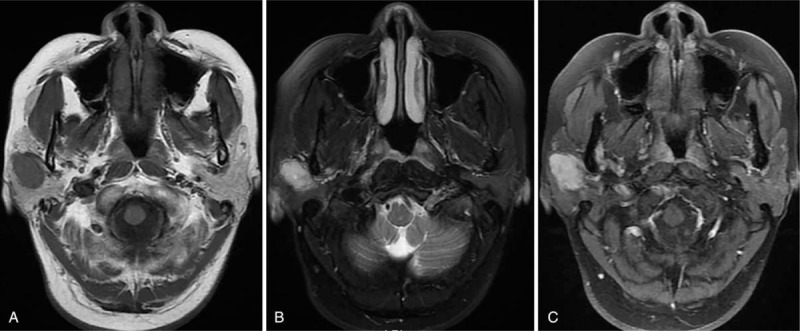
(A and B) The lesion exhibited a solid iso-intensity nodule on T1-weighted images, and high signal intensity with central spot higher signal intensity on T2-weighted fat-suppression images; (C) Axial enhanced images revealing lesion with obvious enhancement and mottled non-enhancement area.

A 48-year-old woman was referred to our hospital with primary complaint of a grape-sized painless mass behind the right ear for 1 month. Physical examination revealed a mass of 1 cm × 2 cm in size in the right parotid gland area. Ultrasonography revealed an oval-shaped hypoecho lesion in the right parotid gland with clear border. Pre-contrast CT images demonstrated the presence of a 2.2 cm × 1.4 cm heterogeneous mass which was iso-density with central lower density and a relatively well-defined margin in the right parotid gland (Fig. 3A). After intravenous injection of contrast material, the mass demonstrated significantly heterogeneous enhancement with central irregular non-enhancement area (Fig. 3B). The CT values of the significant-enhanced areas in pre-contrast and enhanced scans were 44.82 HU and 157.32 HU, respectively. The lesion exhibited a solid iso-signal nodule on T1-weighted images (Fig. 4A), and high signal with central spotty higher signal on T2-weighted fat-suppression images (Fig. 4B). Enhanced images revealed obvious enhancement with mottled non-enhancement area (Fig. 4C).

**Figure 5:**
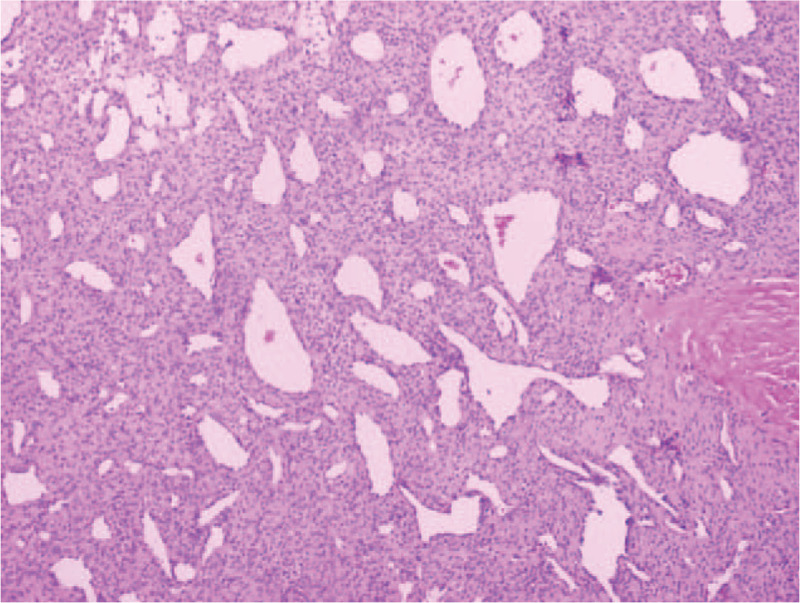
Histopathologic examination showed a proliferation of overlapping plump, spindle-shaped myoid cells in a concentric arrangement, intimately associated with thin-walled vascular channels. (Hematoxylin-eosin stain, original magnification, 100×.)

Excision of the tumor and the superficial parotidectomy with facial nerve preservation were performed. The tumor was between the deep lobe and the superficial lobe with invasion of adjacent tissue. Histopathologic examination showed a proliferation of overlapping plump, spindle-shaped myoid cells in a concentric arrangement, intimately associated with thin-walled vascular channels (Fig. 5). The lesion cells showed positive immunohistochemical reactivity for SMA. Immunostaining was negative for CD34, CD31, desmin, and S-100. The pathologic diagnosis of the surgery specimen was myopericytoma.

The patient had no signs of recurrence during a 5-year follow-up.

## 3 Discussion

Myopericytoma is an uncommon tumor, and reports of myopericytoma in parotid gland are even more rare. Therefore, it is difficult for the surgeons and radiologists to make the accurate diagnosis. Benign myopericytoma is generally considered a slow-growing mass without pain. Myopericytoma usually follows a benign clinical course with local recurrence potential, while malignant myopericytoma has also been reported.^[2–4]^

Histological findings of myopericytoma are characterized by the presence of round or oval-shaped cells with eosinophilic cytoplasm arranged circumferentially around vascular lumina in a multilayered pattern (onion skin), often benign with absence of mitoses, pleomorphism, or necrosis. It is believed that the myopericytoma cell of origin is either the pericyte or myofibroblast, both of which exhibit properties of modified smooth muscle cells rather than endothelial cells.^[7]^ The immunophenotype of the neoplastic cells in myopericytoma is very helpful for the differential diagnosis.^[8]^ The cells of myopericytomas display typically stain positively for SMA and staining for S-100, EMA, CD31, and CD34 is generally negative.^[9]^ The genetic drivers for the development of myopericytoma remain unclear and need future research to better understand etiology, diagnosis, and optimal clinical management for patients with myopericytoma.^[10]^

We reviewed all the previously published cases of parotid gland myopericytoma (Table 1). Previous study suggests that the diameter of myopericytoma is usually less than 2 cm.^[9]^ However, the maximum diameters of all 7 lesions in the 5 published studies and present report of myopericytoma arising from the parotid gland were 2.5 cm, 5.6 cm, 1.4 cm, 1.2 cm, 0.9 cm, 3.0 cm, and 2.2 cm respectively,^[9,11–14]^ which is different from the previous research. The lesion with maximum diameters of 5.6 cm is the recurrent focus not long after the last operation and another lesion with maximum diameters of 3.0 cm is with hemorrhage and necrosis. We speculates that recurrence, hemorrhage, and necrosis might be the cause of the enlarged tumor lesion.

**Table 1:** Literature review for myopericytoma arising from the parotid gland.

Lesion	Age (yr)	Sex	Location	Maximum diameters	Surgical treatment	Follow-up	Reference
1	40	Female	Left parotid gland	2.5 cm	Excision of the tumor and superficial parotidectomy	NS	^[9]^
2	43	Female	Right parotid gland	5.6 cm	Excision of the tumor and total parotidectomy	R	^[11,12]^
3	65	Male	Left parotid gland	1.4 cm	Excision of the tumor and total parotidectomy	2 yr, NR	^[13]^
4	65	Male	Left parotid gland	1.2 cm	Excision of the tumor and total parotidectomy	2 yr, NR	^[13]^
5	66	Male	Right parotid gland	0.9 cm	Extracapsular dissection	18 mo, NR	^[14]^
6	62	Male	Left parotid gland	3.0 cm	Excision of the tumor and superficial parotidectomy	17 mo, NR	Present case
7	48	Female	Right parotid gland	2.2 cm	Excision of the tumor and superficial parotidectomy	5 yr, NR	Present case

NR = no recurrence, NS = not sure, R = recurrence.

Three CT examinations of myopericytoma involving parotid gland showed strong peripheral enhancement and irregular central non-enhanced region after contrast medium administration,^[9,11,12]^ which was similar to the appearance of case two in present report. MR findings were reported by only 1 case with myopericytoma in the parotid gland. Kuczkowski et al reported a case of myopericytoma in the parotid gland showed 2 foci in the left parotid gland with significant heterogeneous enhancement.^[13]^ Myopericytoma arising from other regions also most often appear as an enhanced T1 hypointense, T2 hyperintense mass.^[10]^ The second case in present report had a significant enhancement with mottled non-enhancement area as well. We speculated that the obvious enhancement with or without hemorrhage and necrosis may be the imaging feature for myopericytoma of parotid gland. However, the first case in present report was not enhanced, which is quite different from previous findings. We suppose that this may be related to the hemorrhage in the lesion. To the best of our knowledge, the present report is the first to report hemorrhage in parotid gland myopericytoma. Thus, when a parotid gland mass with a well-defined cyst-solid imaging features and hemorrhage was detected, the diagnosis of myopericytoma should be considered in the differential diagnosis. However, future studies are still needed to verify this guess.

The common MRI features of these 2 cases were cyst-solid mixed appearance, and relatively smoothed margins with hyperintense on T2-weighted image in the solid component. We are the first to provide a comprehensive radiological description of parotid gland myopericytoma, including CT and MRI findings.

The differential diagnosis should include tumors with similar MR findings that are commonly seen in the parotid gland. Warthin's tumors usually demonstrated a rounded or lobulated lesion with intermediate signal on both T1-weighted images and T2-weighted images, and focal T2-hyperintense area corresponding to the cystic components. However, the tumor usually shows rapid contrast enhancement and washout on post-contrast T1-weighted images.^[15]^ The characteristics of pleomorphic adenoma are hyperintense areas on T2-weighted images with marked enhancement, fibrous capsules, and a lobulated contour. Cystic change and hemorrhage are often seen in larger tumors (>3 cm).^[16]^ Basal cell adenoma shows relatively low signal intensity on T1-weighted images and hypointense to slightly hyperintense on T2-weighted images, with rapid and prolonged enhancement on dynamic scans. Basal cell adenoma sometimes has cystic or hemorrhagic components.^[17]^ Mucoepidermoid carcinoma may be predominantly cystic or mixed cystic with solid mural components. The MRI findings have a tendency to be related to the histological grade.^[18]^ There are some general features suggesting malignancy, such as irregular margins, extra-glandular infiltration, perineural spread, and secondary lymphadenopathies.^[15]^

Myopericytoma usually follows a benign clinical course, in our case, after 17-month and 5-year follow-up, both cases had no signs of recurrence, but local recurrence and malignant myopericytoma have also been reported.^[2–4]^ It is generally accepted that parotid gland myopericytoma should be treated with wide surgical excision to prevent local recurrence. The superficial parotidectomy with facial nerve preservation is known as a widely acceptable procedure. Meanwhile, previous study suggests that via extracapsular dissection may provide improved functional and facial aesthetic outcomes for parotid neoplasms compared with superficial parotidectomy.^[14]^ Radiation has been used in selective cases to decrease the likelihood of recurrence, especially if there has been incomplete tumor excision.^[19]^ Ongoing molecular studies may offer more suitable options for patients with malignant myopericytoma in the future.

## 4 Conclusion

To the best of our knowledge, this is the first report to specify the MRI features of myopericytoma involving the parotid gland. Myopericytoma should be considered during the differential diagnosis of painless, cyst-solid mixed lesions with relatively smoothed margins, high signal intensity on T2-weighted images, and obvious enhancement tumor involving parotid gland. It is worth noting that the tumor may present unenhanced mass due to hemorrhage sometimes.

## Acknowledgments

We thank Xiao-Pei Xu for her excellent help in the preparation of the manuscript.

## Author contributions

**Conceptualization:** Yao Pan, Lu Chen, Ri-Sheng Yu.

**Data curation:** Yao Pan, Lu Chen, Dan Shi.

**Formal analysis:** Dan Shi, Ying Chen.

**Methodology:** Yao Pan, Lu Chen, Ri-Sheng Yu.

**Resources:** Yao Pan, Lu Chen, Dan Shi, Ying Chen, Ri-Sheng Yu.

**Supervision:** Ying Chen, Ri-Sheng Yu.

**Writing – original draft:** Yao Pan, Lu Chen.

**Writing – review & editing:** Ying Chen, Ri-Sheng Yu.
